# Correction: Post-transcriptional regulation of MEK-1 by polyamines through the RNA-binding protein HuR modulating intestinal epithelial apoptosis

**DOI:** 10.1042/BCJ20091459_COR

**Published:** 2026-05-21

**Authors:** Jian-Ying Wang, Peng-Yuan Wang, Jaladanki N. Rao, Tongtong Zou, Lan Liu, Lan Xiao, Ting-Xi Yu, Douglas J. Turner, Myriam Gorospe, Jian-Ying Wang

**Affiliations:** Cell Biology Group, Department of Surgery, University of Maryland School of Medicine, Baltimore, MD 21201, U.S.A.

**Keywords:** 3′-untranslated region (3′-UTR), mitogen-activated protein kinase kinase-1 (MEK-1), mRNA stability, ornithine decarboxylase, polyamine, ribonucleoprotein, translational regulation

The authors of the original article “Post-transcriptional regulation of MEK-1 by polyamines through the RNA-binding protein HuR modulating intestinal epithelial apoptosis” (DOI: 10.1042/BJ20091459) would like to correct Figure 8B. Similarities within Figures 7B and 8B were noted by a reader, which included between:

- A region of the Figure 7Bc A-V Staining panel and a region of the Figure8Ba A-V Staining panel
- A region of the Figure8Bb No-TNFa/CHX panel and the Figure8Bd No-TNFa/CHX panel

The authors explained that the Figure 7Bc and the Figure8Bb images were correct, but the wrong images were mistakenly used for the Figure 8B panels a and d during final figure preparations of Figure 8. In addition, the authors provided raw data. The correction has been assessed and approved by the Editorial Board. The authors apologise for this error and declare that these corrections do not change the results or conclusions of their paper. The corrected version of Figure8B is presented here:

**Figure 8B:**
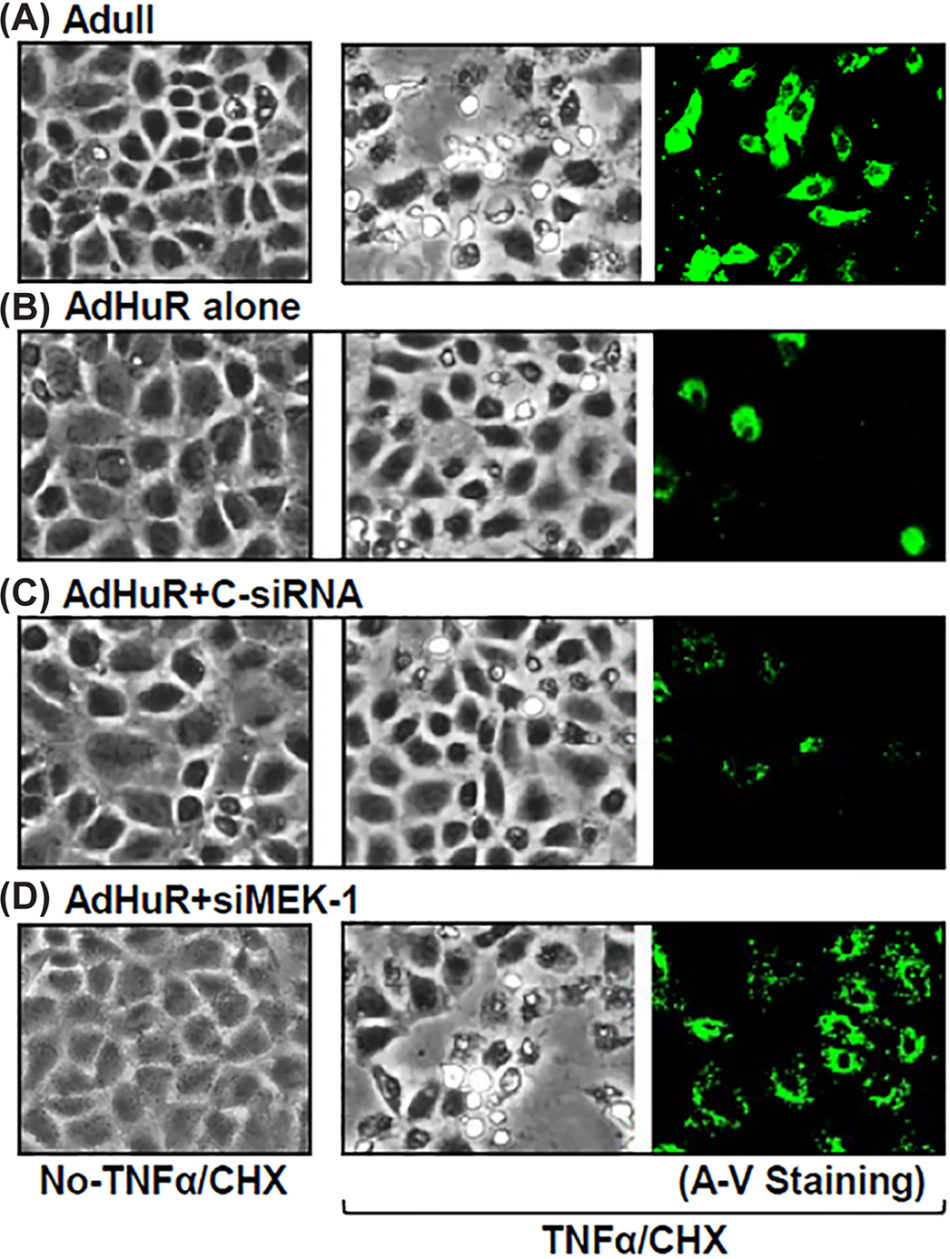
TNFα and CHX-induced apoptosis in cells after different treatments (**A**) cells infected with Adull. (**B**) cells infected with AdHuR. (**C**) cells infected with AdHuR and transfected with C-siRNA. (**D**) cells infected with AdHuR and transfected with siMEK1. Apoptosis was measured by morphological analysis (middle images) and ApoAlert annexin-V (right-hand images) 4 h after treatment with TNFα and CHX. P-Y Wang et al., Biochem J (2010)

